# The mechanisms and treatment of asphyxial encephalopathy

**DOI:** 10.3389/fnins.2014.00040

**Published:** 2014-02-27

**Authors:** Guido Wassink, Eleanor R. Gunn, Paul P. Drury, Laura Bennet, Alistair J. Gunn

**Affiliations:** Fetal Physiology and Neuroscience Team, Department of Physiology, Faculty of Medical and Health Sciences, University of AucklandAuckland, New Zealand

**Keywords:** asphyxia, encephalopathy, hypothermia, neuroprotection, injury

## Abstract

Acute post-asphyxial encephalopathy occurring around the time of birth remains a major cause of death and disability. The recent seminal insight that allows active neuroprotective treatment is that even after profound asphyxia (the “primary” phase), many brain cells show initial recovery from the insult during a short “latent” phase, typically lasting approximately 6 h, only to die hours to days later after a “secondary” deterioration characterized by seizures, cytotoxic edema, and progressive failure of cerebral oxidative metabolism. Although many of these secondary processes are potentially injurious, they appear to be primarily epiphenomena of the “execution” phase of cell death. Animal and human studies designed around this conceptual framework have shown that moderate cerebral hypothermia initiated as early as possible but before the onset of secondary deterioration, and continued for a sufficient duration to allow the secondary deterioration to resolve, has been associated with potent, long-lasting neuroprotection. Recent clinical trials show that while therapeutic hypothermia significantly reduces morbidity and mortality, many babies still die or survive with disabilities. The challenge for the future is to find ways of improving the effectiveness of treatment. In this review, we will dissect the known mechanisms of hypoxic-ischemic brain injury in relation to the known effects of hypothermic neuroprotection.

## Introduction

Moderate to severe neonatal hypoxic-ischemic encephalopathy occurs in approximately 1–3 cases per 1000 term live births in developed nations (Kurinczuk et al., 2010), and remains a significant cause of death and long-term neurodevelopmental disability (Lawn et al., 2005; Marlow et al., 2005). The most devastating complication in survivors is cerebral palsy, which is associated with one of the very highest indices of burden of disease from loss of potential productive members of society and direct burdens on the individual, family, and social institutions that last the entire life (Centers for Disease Control and Prevention (CDC), 2004). Approximately 15% of all cases of cerebral palsy are associated with acute brain damage (encephalopathy) in term infants (Mcintyre et al., 2011).

There is now compelling clinical evidence from meta-analyses of large randomized controlled trials that prolonged moderate cerebral hypothermia in term infants with moderate to severe hypoxic-ischemic encephalopathy, started within a few hours after birth and continued until resolution of the acute phase of delayed cell death improves neurodevelopmental outcome in the medium to long-term (Edwards et al., 2010; Guillet et al., 2012; Shankaran et al., 2012b). This functional improvement is consistent with reduced brain injury on modern imaging (Rutherford et al., 2010; Shankaran et al., 2012a). Hypothermia suppresses many potentially deleterious mechanisms, making it difficult to distinguish between physiological changes during cooling that are critically beneficial, compared with those that are either indifferent or even deleterious.

Current therapeutic hypothermia protocols are incompletely neuroprotective, reducing the combined risk of death and severe disability at 18 months of age by approximately 11% (Edwards et al., 2010). Thus, many children continue to die, or survive with moderate to severe handicap despite treatment with current hypothermia protocols. Expanding our knowledge of the key therapeutic targets of hypothermia could help to further improve existing protection. In the present review we will critically assess the potential mechanisms of hypothermic neuroprotection in relation to the known window of opportunity for cooling after severe hypoxia-ischemia (HI).

## The evolution of hypoxic-ischemic injury

**Figure 1:**
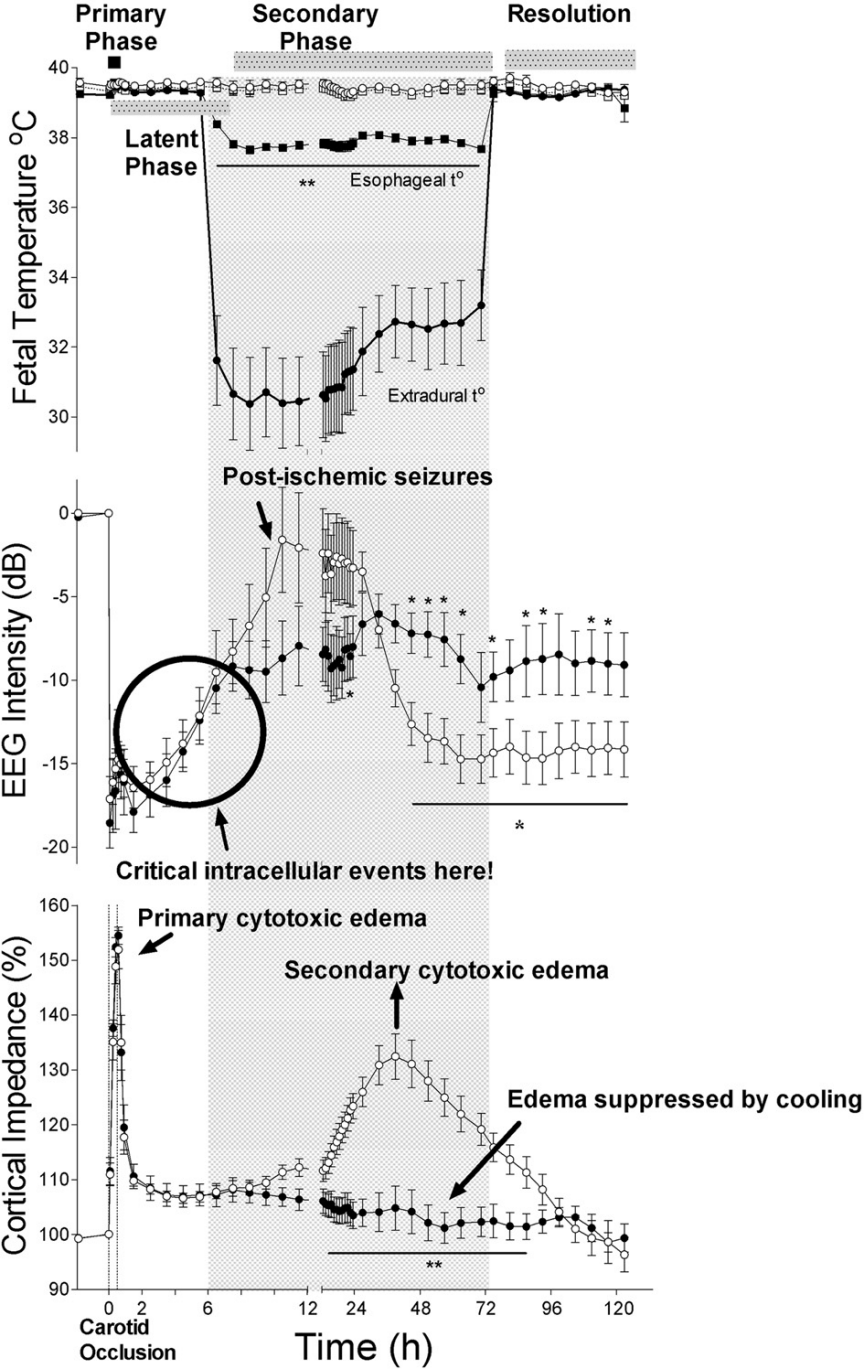
**The effects of 30 min of cerebral ischemia (from *t* = 0, shown by vertical dotted lines) with or without induced mild hypothermia (shown by the bar) started 5.5 h after reperfusion in near-term fetal sheep on fetal temperature, electroencephalographic (EEG) intensity and cortical impedance (a measure of cell swelling).** The top panel shows changes in extradural (solid circles) and esophageal (solid squares) temperature in the hypothermia group and extradural (open circles) and esophageal (open squares) temperature in the normothermia group. The lower two panels show changes in EEG intensity (dB) and cortical impedance (expressed as percentage of baseline) in the hypothermia (solid circles) and normothermia (open circles) groups. The hypothermia group showed greater recovery of EEG intensity after resolution of delayed seizures and complete suppression of the secondary rise in impedance. Data modified from Gunn et al. (1998). ^*^p <0.05; ^**^p <0.001.

**Figure 2:**
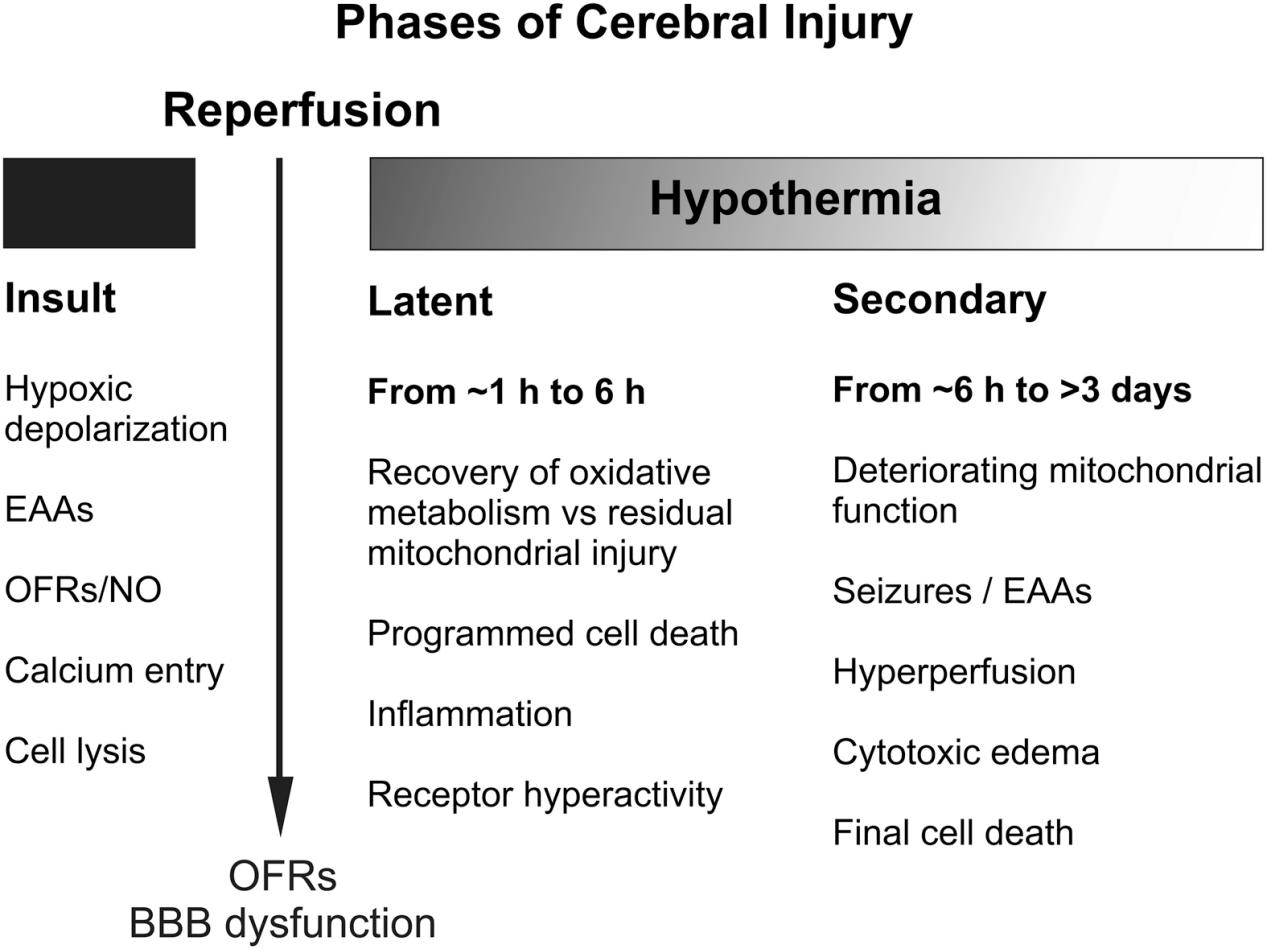
**Schematic diagram illustrating the different pathological phases of cerebral injury after severe hypoxic-ischemia.** OFRs, oxygen free radicals; BBB, blood brain barrier; EAAs, excitatory amino acids; NO, nitric oxide.

The central insight that underpinned development of therapeutic hypothermia was that HI injury evolves over time, as illustrated in Figure 1 (Gunn and Thoresen, 2006). We now know that although some neurons likely die during the actual ischemic or asphyxial event (the “primary” phase of injury, Figures 1, 2), the hypoxia-induced impairment of cerebral oxidative metabolism, cytotoxic edema, and accumulation of excitatory amino acids (EAAs) typically recovers at least partially over approximately 30–60 min (Tan et al., 1996; Bennet et al., 2007b). This is followed by a “*latent*” phase during which electroencephalogram (EEG) activity remains suppressed but high energy phosphates are normal or near normal (Azzopardi et al., 1989; Gunn et al., 1997; Bennet et al., 2006; Iwata et al., 2008). Notably, during this phase there is post-asphyxial cerebral hypoperfusion associated with reduced cerebral metabolism and *improved* tissue oxygenation in the near-term lamb (Jensen et al., 2006), consistent with active suppression of cerebral metabolism.

With moderate to severe injury, this period is frequently followed by progressive secondary deterioration many hours later (from approximately 6–15 h), that is typically associated with stereotypic seizures (Gunn et al., 1997), cytotoxic edema and accumulation of excitotoxins, near-complete failure of cerebral mitochondrial activity (Lorek et al., 1994; Bennet et al., 2006) and eventual spreading cell death. More severe insults are typically associated with evidence of greater primary damage (Williams et al., 1992), and earlier and more severe secondary deterioration and neuronal loss (Williams et al., 1992; Lorek et al., 1994; Sabir et al., 2012). In turn, the severity of secondary oxidative metabolism derangement in newborns with birth asphyxia is strongly associated with increased risk of mortality and adverse neurodevelopmental outcome (Roth et al., 1992).

## Mechanisms of injury during hypoxia-ischemia

Brain injury requires a period of insufficient delivery of oxygen and substrates such as glucose (and lactate in the fetus) such that neurons and glia cannot maintain homeostasis. Once the neuron's supply of high-energy metabolites such as adenosine triphosphate (ATP) can no longer be maintained during HI, the energy dependent mechanisms of intracellular homeostasis including the Na^+^/K^+^ ATP dependent pump begin to fail. Neuronal depolarization occurs, leading to sodium and calcium entry into cells. This creates an osmotic and electrochemical gradient that in turn favors further cation and water entry leading to cell swelling (cytotoxic edema). Consequently, this may lead to acute cell lysis when the homeostatic disturbance is sufficiently severe (Rothman and Olney, 1995). Even after surprisingly prolonged and severe insults, however, many swollen neurons still recover, at least temporarily, if the hypoxic insult is reversed or the osmotic environment is manipulated. Evidence suggests that several additional factors act to increase cell injury during and following depolarization. One of these factors is the extracellular accumulation of EAAs after neuronal depolarization coupled with impaired energy dependent re-uptake, which promote further receptor mediated cell swelling and intracellular calcium entry (Rothman and Olney, 1995). Another factor is the generation of oxygen free radicals such as the highly toxic hydroxyl radical (▪OH), leading to lipid peroxidation and DNA/RNA fragmentation (Bagenholm et al., 1998; Fraser et al., 2008). Further, there is compelling evidence that generation of the reactive oxygen species nitric oxide (NO▪) by neuronal nitric oxide synthase (nNOS) is increased during and after HI, and that reaction of NO▪ with superoxide in the cytosol and mitochondria produces peroxynitrite and other reactive nitrogen species that are associated with cell membrane, organelle and mitochondrial damage (Tan et al., 1999). For example, selective nNOS blockade during asphyxia reduced neuronal damage in the basal ganglia of preterm fetal sheep (Drury et al., 2013). However, these factors largely appear to be injurious only in the presence of hypoxic cell depolarization.

### Hypothermia during hypoxia-ischemia

Hypothermia produces a reduction in cerebral metabolism of approximately 5% for every 1 degree fall in temperature (Laptook et al., 1995), which delays the onset of anoxic cell depolarization. The protective effects of hypothermia are not simply due to reduced metabolism alone, however, since graded cooling substantially reduces damage for a given absolute duration of depolarization compared to normothermia (Bart et al., 1998). The reduced accumulation of EAAs during intra-ischemic hypothermia (Nakashima and Todd, 1996a; Ooboshi et al., 2000), is primarily due to delay in depolarization, although there is some evidence for a reduction in the rate of release even after depolarization has occurred (Nakashima and Todd, 1996b). In addition, hypothermia potently suppresses NO▪ and superoxide formation in hippocampal slice cultures (Mcmanus et al., 2004), during ischemia and reperfusion in rodents (Lei et al., 1997), cardiac arrest in young adult dogs (Lei et al., 1994), and during and immediately after HI in the piglet (Thoresen et al., 1997).

### Hypothermia during reperfusion

Once blood flow and oxygenation are restored after acute HI, there is a rapid burst of NO▪ and superoxide formation (Thoresen et al., 1997), and EAA levels rapidly fall in parallel with resolution of the acute cell swelling, typically over 30–60 min (Tan et al., 1996; Fraser et al., 2008). It would appear that this recovery can be accelerated by cooling; hypothermia started immediately after HI in newborn piglets was associated with reduced extracellular levels of EAAs, and reduced NO▪ efflux in the brain (Thoresen et al., 1997).

### Hypothermia and the blood brain barrier

Ischemic brain injury is generally associated with marked opening of the blood brain barrier (BBB) which may contribute to further brain swelling. In adult rats, cooling after global ischemia was associated with reduced BBB leakiness and brain edema 24 h later, but only when induced within 1 h after ischemia (Preston and Webster, 2004; Baumann et al., 2009). Such stabilization of the BBB by hypothermia was associated with attenuated degradation of key regulatory proteins in the vascular basement membrane (Baumann et al., 2009). Similarly, hypothermia induced 1 h after focal ischemia in rats was associated with improved BBB function, possibly by inhibiting metalloproteinase induction (Nagel et al., 2008). However, the inhibition of metalloproteinases after HI in neonatal rats has had inconsistent effects (Ranasinghe et al., 2012). Given that treatment with hypothermia is highly neuroprotective even when delayed by more than an hour after HI (Gunn et al., 1997, 1998; Colbourne et al., 2000; Sabir et al., 2012), it seems unlikely that this mechanism is a major contributor to hypothermia's beneficial effects.

## Critical clues from studies of hypothermia during the latent phase

Although it remains unclear when exactly the brain becomes irreversibly injured, there are consistent empirical data that the so-called “latent” or early recovery phase of transient restoration of cerebral oxidative metabolism, before onset of secondary energy failure, represents the most realistic window of opportunity for therapeutic intervention (Roelfsema et al., 2004; Gunn and Gluckman, 2007). Thus, pragmatically, the window of intervention appears to close indefinitely during secondary energy failure, which corresponds either to overt cell death (Vannucci et al., 2004), or to a critical “irreversible” stage in the evolution of delayed cell death (Gunn and Gluckman, 2007).

For example, in the near-term fetal sheep, moderate hypothermia induced 90 min after reperfusion from a severe episode of cerebral ischemia (i.e., in the early latent phase) and continued until 72 h after ischemia, prevented secondary cytotoxic edema, improved electroencephalographic recovery, and reduced neuronal and white matter injury (Gunn et al., 1997; Roelfsema et al., 2004). However, only partial protection was seen in this paradigm when hypothermia was delayed until just before the onset of secondary seizures (5.5 h after reperfusion, e.g. see Figure 1) (Gunn et al., 1998; Roelfsema et al., 2004), and there was no significant protection when the start of cooling was delayed until after seizures were established (8.5 h after reperfusion) (Gunn et al., 1999).

These observations are consistent with previous studies in anaesthetized piglets subjected to severe HI, where application of prolonged moderate to deep hypothermia for 24 h immediately after resuscitation prevented secondary energy failure, suppressed seizure burden, and reduced neural loss in the cortex and basal ganglia (Edwards et al., 1995; Thoresen et al., 1995; Tooley et al., 2003). Similar progressive loss of neuroprotection was found when 5 h of moderate hypothermia was delayed for up to 6 h after moderate HI in postnatal day 7 rat pups (Sabir et al., 2012), while this relatively short period of hypothermia was not protective at all after a more severe insult. Studies in adult rodents strongly suggest that more prolonged cooling, for 48 h or more, further improves the effect of delayed cooling (Colbourne et al., 1999a); it remains unclear whether there would be further benefit if cooling was continued for longer than approximately 48–72 h.

### Evolution of injury during the latent phase

The precise mechanism(s) which initiate the cascade leading to delayed cell death after HI remain incompletely understood, but are undoubtedly multi-factorial with excessive calcium influx, pro- and anti-apoptotic proteins, and trophic factor withdrawal during and after HI all playing a critical role (Zipfel et al., 2000; Hagberg et al., 2009). For example, neural accumulation of calcium precipitates continues for many hours after HI in the immature rodent (Stein and Vannucci, 1988), and is associated with worsening structural morphology (Puka-Sundvall et al., 2000). Although mitochondria can buffer increased levels of neuronal cytosolic calcium (Wang and Thayer, 1996), excessive sequestration of calcium by mitochondria may lead to inhibition of the respiratory chain, uncoupling of oxidative phosphorylation (Szydlowska and Tymianski, 2010), and permeabilization of the mitochondrial membranes (the “intrinsic” apoptosis pathway).

**Figure 3:**
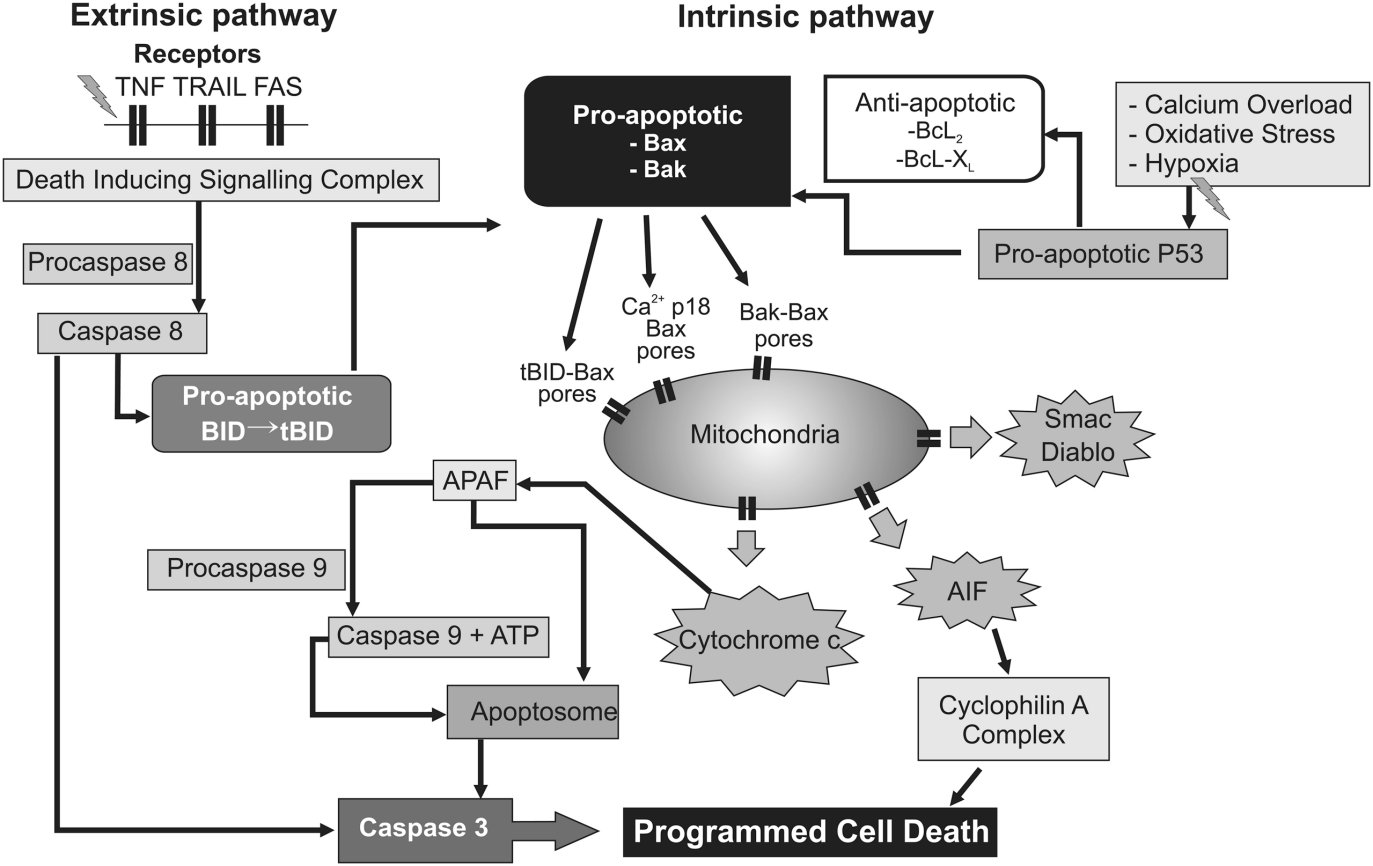
**Flow chart depicting several intracellular mechanisms associated with permeabilization of the mitochondrial membranes, leading to progressive failure of mitochondrial oxidative phosphorylation and ultimately delayed programmed cell death.** Upstream triggers such as inflammation and trophic withdrawal activate cell surface death receptors initiating the “extrinsic” pathway to programmed cell death. Conversely, calcium overload and oxygen free radicals appear to exert their effect predominantly at the mitochondrial level via the “intrinsic” pathway. In addition, cross-over activation between the “extrinsic” and “intrinsic” pathway may take place through pro-apoptotic intermediates such as the BID protein. AIF, apoptosis inducing factor. Apaf-1, apoptotic protease-activating factor−1; ATP, adenosine triphosphate; BAK, Bcl_2_-antagonist/killer 1; BAX, Bcl_2_-associated × protein; Bcl_2_, B-cell lymphoma 2 protein family; Bcl-X_*L*_, B-cell lymphoma-extra-large; BID, BH3 interacting-domain death agonist; Diablo, direct inhibitor of apoptosis binding protein with low Pi; P53, p53 tumor suppressor protein; Smac, Second mitochondria-derived activator of caspase; tBID, truncated BH3 interacting-domain death agonist; TNF, tumor necrosis factor receptor; TRAIL, TNF-related apoptosis-inducing ligand receptor.

In detail, HI results in activation of pro- and anti-apoptotic proteins (Hagberg et al., 2009), and translocation of cytosolic Bcl-2 associated × protein (Bax) to mitochondria (Rousset et al., 2012), where it oligomerizes (with Bcl-2 family members Bak and Bid) and creates non-specific protein pores in the outer mitochondrial membrane. This allows leakage of pro-apoptotic proteins and cytochrome c into the cytosol (Figure 3) (Hagberg et al., 2009). Not only are pro- and anti-apoptotic proteins and caspase-3 highly expressed in the immature brain (Polster et al., 2003; Zhu et al., 2005; Soane et al., 2008), but regulating their interaction can provide significant neuroprotection after perinatal HI (Gibson et al., 2001; Wang et al., 2010; Nijboer et al., 2011). As the terminal electron acceptor of the mitochondrial electron transport chain, loss of cytochrome c oxidase may be particularly decisive in the uncoupling of oxidative phosphorylation, since it accounts for over 90% of cellular oxygen consumption (Cooper and Springett, 1997). Thus, the permeabilization of mitochondrial membranes, with detachment of soluble protein cytochrome c from cardiolipin on the inner mitochondrial membrane (Hagberg et al., 2009), and release of apoptogenic factors including second mitochondria-derived activator of caspase (Smac), direct inhibitor of apoptosis binding protein with low Pi (Diablo) and apoptosis inducing factor (AIF) from the mitochondrial intermembrane space (Martinou and Green, 2001), could shift a potentially reversible injurious paradigm to irrevocable cell death.

After its release cytosolic cytochrome c binds to apoptotic protease-activating factor-1 (Apaf-1), and activates caspase 9 in the presence of ATP or dATP to form an apoptosome, which initiates the executioner caspase-3 (Martinou and Green, 2001), and ultimately leads to DNA fragmentation (Figure 3). This functional decline of mitochondria appears to be consistent with the progressive fall in cytochrome oxidase seen from 3 h onwards after asphyxia in fetal sheep (Bennet et al., 2010), before onset of secondary cytotoxic edema and stereotypic seizures. Critically, one kinetic study using HeLa cell lines showed that, after initiation of cytochrome c release, all cytochrome c was invariably released from mitochondria within 5 min, and this release preceded the apoptotic cascade (Goldstein et al., 2000). There is good histological evidence that programmed cell death is a significant contributor to post-hypoxic cell death in the developing human brain (Edwards et al., 1997; Scott and Hegyi, 1997). Additional factors that likely help trigger or augment programmed cell death include loss of trophic support from astrocytic growth factors (Clawson et al., 1999), and the inflammatory reaction to HI (Giulian and Vaca, 1993), through activation of cell surface death receptors (and thus the “extrinsic” apoptosis pathway; Graham et al., 2004). The delayed increase in synthesis of down-stream mediators of cell death such as NO and reactive oxygen species in this phase are likely secondary to inflammation (Gehrmann et al., 1995). In addition, recent data indicate that “extrinsic” TNF and Fas death receptor engagement can also promote cross-over activation of the “intrinsic” apoptotic pathway through association with the pro-apoptotic tBid protein on Bak and Bax (Figure 3) (Wei et al., 2001; Schug et al., 2011).

### Does hypothermia specifically suppress delayed programmed cell death?

There is evidence that hypothermia may have a particular role in suppressing the evolution of programmed cell death. Studies using morphological criteria have had mixed outcomes. In the piglet, hypothermia started after severe HI was reported to reduce apoptotic cell death, but not necrotic cell death (Edwards et al., 1995), with similar results reported after injury in rats (Xu et al., 1998; Inamasu et al., 2000). However, in the adult rat, delayed post-ischemic cell death prevented by hypothermia had a necrotic appearance on detailed electron microscopic criteria (Colbourne et al., 1999b), consistent with findings of a maturity related reduction in cytochrome c release, AIF and caspase-3 expression after HI in rodents (Hu et al., 2000; Zhu et al., 2005).

It is important to appreciate that in practice these morphological changes often do not closely reflect the underlying pathways leading to cell death. By analogy with the active process of developmental “pruning” of excess cells (including neurons), it was originally suggested that an apoptotic morphology reflected activation of “pre-programmed” cell death pathways (Beilharz et al., 1995; Dell'anna et al., 1997). Although necrosis can reflect immediate biophysical damage to the cell, such as membrane instability or ion shifts leading to cell lysis in the primary phase (Beilharz et al., 1995), there is now compelling evidence that post-hypoxic cell death represents a continuum between apoptosis and necrosis, as recently reviewed (Northington et al., 2011). *In vitro*, hypothermia during severe hypoxia reduced both “apoptotic” and “necrotic” morphological cell death in developing rat neurons, and suppressed hypoxia-associated protein synthesis (Bossenmeyer-Pourie et al., 2000). Microarray analysis in rats after focal ischemia confirmed that post-insult hypothermia suppressed gene responses to ischemia, particularly genes involved in calcium homeostasis, cellular and synaptic integrity, inflammation, cell death, and apoptosis (Nagel et al., 2012).

Although there are multiple pathways to programmed cell death, as discussed above, caspase-3 is the final “executioner” caspase, and thus caspase-3 activation may be used as a reasonable marker of these pathways. Other *in vitro* studies have shown that mild hypothermia suppressed H_2_*O*_2_-induced apoptosis and caspase-3 induction in rat cortical neurons (Li et al., 2012), and suppressed neuronal apoptosis induced by serum deprivation, with significantly reduced activation of caspases -3, -8, and -9 after 24 h, and reduced cytochrome c translocation consistent with suppression of both the intrinsic and extrinsic pathways of apoptosis (Xu et al., 2002). Further, hypothermia during focal ischemia in adult rats reduced expression of the cell death receptor Fas and activation of caspase-8, supporting a direct effect on the extrinsic pathway of apoptosis (Liu et al., 2008).

These studies examined forms of *intra-insult* cooling. However, there is supporting evidence from post-insult cooling *in vivo*. For example, in the near-term fetal sheep, hypothermia delayed for 90 min after ischemia markedly suppressed caspase-3 activation in white matter (Roelfsema et al., 2004). Similarly, in postnatal day 7 (P7) rats, an age when brain development is comparable to the late preterm human infant (Rice et al., 1981), immediate induction of hypothermia after HI reduced caspase-3 expression in the core area of cortical infarction but not in the penumbra (Askalan et al., 2011), suggesting that hypothermia modulated both caspase-dependent and independent mechanisms. In the same paradigm, hypothermia also reduced caspase-3 activation in pre-oligodendrocytes (Xiong et al., 2013). In adult rats, mild hypothermia (33°C) for 2 h after transient endothelin-induced focal ischemia suppressed activated caspase-3 immunoreactivity up to a week after the insult (Zgavc et al., 2013). Similarly, hypothermic neuroprotection after transient global ischemia in adult rats was associated with upregulation of the anti-apoptotic protein bcl-2, and reduced expression of the pro-apoptotic protein p53 (Zhang et al., 2010).

There is some evidence from adult models that hypothermia can specifically suppress the mitochondrial permeability transition. For example, mild hypothermia either during or shortly after transient focal ischemia attenuated the release of cytochrome c (Yenari et al., 2002; Zhao et al., 2004). Similarly, protection of the CA1 region of the hippocampus with mild hypothermia after global ischemia was associated with reduced cytochrome c release after 48 h, and with reduced caspase-3 and -9 activity at 12 and 24 h (Zhao et al., 2005). Further, neuroprotection with immediate, prolonged cooling after cardiac arrest in adult minipigs was associated with suppression of mitochondrial permeability pore opening, leading to reduced release of cytochrome c and other pro-apoptotic factors including AIF and reduced caspase-3 activation (Gong et al., 2013). Post-ischemic hypothermia maintained mitochondrial respiratory activity 2 h after reperfusion in the adult gerbil (Canevari et al., 1999), and intra-ischemic hypothermia has been shown to preserve activity for 4 days after a similar insult in neonatal rats (Nakai et al., 2001). Presently, there are no data evaluating mitochondrial function *in vivo* during therapeutic hypothermia following HI in the developing brain. However, hypothermia after cardiac arrest in adult minipigs substantially improved recovery of mitochondrial membrane potential and respiration (Gong et al., 2013).

Finally, there is evidence that combined treatment with the anti-apoptotic agent, insulin-like growth factor 1 (Guan et al., 2001), and hypothermia starting 4.5 h after cerebral ischemia in near-term fetal sheep did not show additive neuroprotection (George et al., 2011). This observation suggests that their mechanisms of action overlap. Taken as a whole, these studies indicate that hypothermia can suppress apoptosis, likely through several pathways including reduction of mitochondrial permeability (the intrinsic pathway) and reduced activation of the extrinsic pathway of apoptosis.

### Hypothermia and inflammatory second messengers

Brain injury leads to induction of the inflammatory cascade with increased release of cytokines and interleukins (IL) (Hagberg et al., 2012). These compounds are believed to exacerbate delayed injury, whether by direct neurotoxicity and induction of apoptosis or by promoting leukocyte diapedesis into the ischemic brain. Experimentally, cooling potently suppresses this inflammatory reaction (Roelfsema et al., 2004). For example, *in vitro*, hypothermia inhibits microglia proliferation, chemotaxis, induction of pro-inflammatory cytokines, and attenuates microglia neurotoxicity, during and critically, after exposure to both hypoxia and lipopolysaccharide (Si et al., 1997; Schmitt et al., 2007; Seo et al., 2012). Further, hypothermia can suppress translocation and binding of nuclear factor kappa-B, a key inflammatory transcription factor that is activated after cerebral ischemia (Yenari and Han, 2006). In addition, there is limited evidence that cooling microglia after activation with lipopolysaccharide increases production of anti-inflammatory cytokines in culture (Diestel et al., 2010).

Consistent with these *in vitro* findings, hypothermia in adult rats after transient focal ischemia reduced delayed increases in interleukin-1β (IL-1β) and tumor necrosis factor alpha (TNF-α) but not transforming growth factor beta (TGF-ß) in the striatum (Ceulemans et al., 2011). In adult pigs, immediate mild cooling after brief cardiac arrest was associated with attenuation of IL-1β, TNF-α, intercellular adhesion molecule-1 mRNA, and interleukin-1 (IL-1) protein induction 24 h after resuscitation (Meybohm et al., 2010). Similarly, post-insult hypothermia has been associated with consistent suppression of activated microglia after transient ischemia or asphyxia in fetal sheep (Roelfsema et al., 2004; Bennet et al., 2007c; Barrett et al., 2012; George et al., 2012). This broad reduction in inflammatory signaling may offer significant mitochondrial protection. For example, cytokine mediated iNOS expression increases NO▪ levels, which compete with molecular oxygen at its binding site on cytochrome oxidase (Brown, 1997), potently suppressing oxidative metabolism and thus reducing ATP levels (Tatsumi et al., 2000). Furthermore, TNF-α and interferon-γ mediated iNOS expression were associated with mitochondrial DNA damage and apoptosis in cultured oligodendrocytes (Druzhyna et al., 2005).

Intriguingly, despite the potent suppression of microglia by hypothermia, it has little effect on astrocytic proliferation *in vitro* (Si et al., 1997). This raises the possibility that hypothermic protection against post-ischemic neuronal damage may be, in part, the result of differential effects on glia, with suppression of microglial activation but relative sparing of potentially pro-survival astrocytic reactions.

### Hypothermia and excitotoxicity

In contrast to their role during the primary and reperfusion phases, the importance of excitotoxins *after* reperfusion remains surprisingly unclear, given that extracellular levels rapidly return to baseline values (Tan et al., 1996; Thoresen et al., 1997). The apparent protection seen in early studies of anti-excitotoxic agents is difficult to interpret as these studies did not control for cerebral temperature (Mcdonald et al., 1987; Hattori et al., 1989). Indeed, subsequent studies suggested that the effects of glutamate blockade during HI were either largely or synergistically mediated through drug-induced hypothermia (Ikonomidou et al., 1989; Engidawork et al., 2001). In an elegant study in the adult rat, Nurse and Corbett showed that the apparent neuroprotective effect of NBQX, a glutamate antagonist administered from 1 h after mild cerebral ischemia, was directly associated with mild *endogenous* hypothermia for several days that developed an hour after drug administration (Nurse and Corbett, 1996). Strikingly, similar neuroprotection was induced by application of the same hypothermia profile over 28 h, while conversely NBQX “neuroprotection” was abolished by maintaining normothermia. Furthermore, anti-excitotoxin therapy limited to the secondary phase did not reduce neuronal injury in the severely injured parasagittal cortex of fetal sheep, and had only limited neuroprotective effects in more mildly affected areas of the brain (Tan et al., 1992; Gressens et al., 2011).

Nevertheless, even with normal levels of extracellular glutamate, excitotoxicity may still play an indirect injurious role. Pathological hyperexcitability of glutamate receptors has been reported in P10 rats for many hours after HI, with improved neuronal outcome after receptor blockade (Jensen et al., 1998). Further, neuronal death after ischemia has been associated with a selective, delayed change in the composition of the alpha-amino-3-hydroxy-5-methyl-4-isoxazolepropionic acid (AMPA) receptor, with specific down-regulation of GluR2, the subunit that limits Ca^2+^ influx through the AMPA receptor. This may facilitate further excessive influx of Ca^2+^ during spontaneous glutaminergic activity after HI, thus promoting programmed cell death. In adult gerbils, delayed hypothermia from 20 min after global ischemia attenuated these changes in the GluR2 subunit, providing an additional possible mechanism for indirect protection from excessive excitation (Colbourne et al., 2003).

Supporting this hypothesis, despite suppression of overall EEG activity for many hours after asphyxia, transient epileptiform activity was seen in the early recovery phase in preterm sheep fetuses that developed severe injury (George et al., 2004), and was correlated with the severity of neuronal loss in the striatum and hippocampus (Dean et al., 2006b; Bennet et al., 2007c). Suppression of these EEG transients with a glutamate receptor antagonist partially reduced cell loss (Dean et al., 2006a). Furthermore, neuroprotection with post-asphyxial moderate cerebral hypothermia in the preterm fetal sheep was associated with a marked reduction in numbers of epileptiform transients in the first 6 h after asphyxia, and reduced amplitude but not numbers of delayed seizures (Bennet et al., 2007a). The combination of glutamate receptor antagonist infusion and hypothermia after severe asphyxia in preterm fetal sheep, however, showed non-additive neuroprotection, consistent with the suggestion that therapeutic hypothermia is partly protective by attenuating this receptor hyperactivity (George et al., 2012). Further studies are needed to determine whether this is also the case after hypoxic-ischemic injury in the term-equivalent brain.

### Hypothermia and induction of growth factors

Contrary to the assumption that hypothermia generally suppresses new protein synthesis, there is evidence in the adult rat that mild hypothermia after cardiac arrest or ischemia is associated with augmentation of growth factors such as brain-derived neurotrophic factor (BDNF), glial-cell-line derived neurotrophic factor and extracellular-signal regulated kinase (D'Cruz et al., 2002; Schmidt et al., 2003; Kim et al., 2011), which might help protect injured cells. However, BDNF infusions during cardiac arrest in normothermic rats were not neuroprotective (Callaway et al., 2008). Thus, induction of these growth factors alone does not seem to explain the protective effects of mild hypothermia after resuscitation.

### Hypothermia during the secondary phase

Mitochondrial failure is a hallmark of secondary injury evolution (Bennet et al., 2006). Thus, maintaining mitochondria intact after severe HI is crucial in promoting neuroprotection. There is compelling evidence that hypothermia started in the latent phase must be continued for 48 h or more to maintain improved recovery of mitochondrial membrane potential and respiration (Gong et al., 2013), and prevent cell death (Gunn and Thoresen, 2006). The precise reasons are unknown. The most likely explanation is that it is necessary to continue suppressing the programmed cell death and inflammatory pathways until normal homeostasis returns. Alternatively, it could in part reflect suppression of secondary events in this phase, including hyperperfusion, cytotoxic edema and delayed seizures. For example, selective head or whole body cooling in newborn piglets failed to provide any protection when delayed until 3 h after the insult and continued for only 24 h (Karlsson et al., 2008).

In the near-term fetal sheep, cerebral hypothermia started 5.5 h after ischemia and continued until 72 h, was associated with marked extension of secondary hypoperfusion, to nearly 24 h after the insult (Gunn et al., 1998). We have previously shown that this hypoperfusion phase is associated with suppressed cerebral metabolism in the preterm fetal sheep (Jensen et al., 2006). Although prolonged cerebral hypoperfusion after asphyxia neonatorum has been associated with adverse clinical outcome (Elstad et al., 2011), the prolongation of reduced blood flow from cooling was associated with improved neural outcome (Gunn et al., 1998). Conversely, the subsequent onset of secondary hyperperfusion is strongly associated with injury, both in fetal sheep (Bennet et al., 2006), and in newborn infants (Elstad et al., 2011). However, cooling in the fetal sheep prevents hyperperfusion, independently of subsequent neuroprotection (Gunn et al., 1997, 1998).

Similarly, neuroprotection with cerebral cooling started 90 min after cerebral ischemia was associated with potent prevention of secondary cytotoxic edema in near-term fetal sheep (Gunn et al., 1997). Intriguingly, hypothermia started after the onset of secondary seizures (8.5 h after ischemia) also completely prevented secondary cytotoxic edema in the same paradigm, but offered no significant neuroprotection (Gunn et al., 1999). *In vitro*, cooling can prevent intracellular ion and water entry and the consequent osmotic cell swelling even if the ATP dependent Na^+^/K^+^ pump is inhibited by ouabain (Zeevalk and Nicklas, 1996). These findings suggest that suppression of cell swelling is not a direct mechanism of hypothermic neuroprotection.

Clinical studies have found a reduced seizure burden during cooling of infants with hypoxic-ischemic encephalopathy (Glass et al., 2011; Srinivasakumar et al., 2013). Interestingly, despite the effects on cerebral metabolism and perfusion, and the reduction in neuronal loss, post-insult hypothermia did not significantly reduce the rate of electrographic seizures after ischemia or asphyxia in fetal sheep, but did reduce their amplitude (Gunn et al., 1998; Bennet et al., 2007a). Assuming that coupling of flow-metabolism remains intact, the reduced carotid blood flow suggests that cooling may have ameliorated the excessive local metabolic demand associated with seizure activity, which has been shown to directly mediate local neuronal death (Ingvar, 1986; Pereira De Vasconcelos et al., 2002). Treatment with MK-801, a highly potent, selective glutamate antagonist, between 6 and 24 h after cerebral ischemia prevented delayed post-ischemic seizures and completely suppressed fetal EEG activity (Tan et al., 1992). Surprisingly, there was no improvement in parasagittal neuronal loss, and only a modest improvement in less damaged regions such as the temporal lobe (Tan et al., 1992). These findings suggest that severe seizure activity in the secondary phase may contribute to spreading of injury from the core area of damage to more mildly affected regions, and thus anticonvulsant therapy during hypothermia might modulate outcome, although it would be unlikely to alleviate major disabilities.

## Clinical implications?

Collectively, the experimental findings described in this review strongly indicate that to achieve optimal neuroprotection, therapeutic hypothermia should be initiated as soon as possible after resuscitation, within approximately the first 6 h of life, should involve cooling by approximately 3–5°C, and should be continued for approximately 48–72 h until resolution of the secondary phase of injury. Given that in clinical trials to date infants cooling was not started until typically 4.5–5 h (Edwards et al., 2010), it is extremely likely that a pragmatic focus on starting cooling earlier would substantially improve the outcomes of current clinical protocols for therapeutic hypothermia (Thoresen et al., 2013).

Beyond this, further advances are likely to come from developing complementary therapies to add to cooling. As outlined in this review, the protective mechanisms that underlie therapeutic hypothermia are multifactorial. Suppression of inflammation, intracellular signaling and programmed cell death are all important potential pathways to neuroprotection after induced hypothermia. Strategies to augment hypothermic protection may involve protecting mitochondrial function during the latent phase, for example with the natural hormone melatonin (Robertson et al., 2012). Alternatively it may be possible to actively augment neural recovery during long-term recovery after treatment with hypothermia, for example with cell therapy as suggested by preclinical models (Bennet et al., 2012).

### Conflict of interest statement

The authors declare that the research was conducted in the absence of any commercial or financial relationships that could be construed as a potential conflict of interest.
